# Advanced Paclitaxel Delivery Systems in Oncology and Cardiovascular Medicine: Current Formulations and Emerging Technologies

**DOI:** 10.3390/molecules31203652

**Published:** 2026-10-13

**Authors:** Andreea Crețeanu, Claudiu N. Lungu, Eliza Gratiela Popa, Liliana Mititelu Tartau, Alina Diana Panainte, Mihai Creteanu

**Affiliations:** 1 Department of Pharmaceutical Technology, Faculty of Pharmacy, Grigore T. Popa University of Medicine and Pharmacy, 700115 Iasi, Romania; eliza.popa@umfiasi.ro; 2 Department of Functional and Morphological Science, Faculty of Medicine and Pharmacy, Dunarea de Jos University, 800010 Galati, Romania; 3 Department of Pharmacology, Faculty of Medicine, Grigore T. Popa University of Medicine and Pharmacy, 700115 Iasi, Romania; liliana.tartau@umfiasi.ro; 4 Department of Analytical Chemistry, Faculty of Pharmacy, Grigore T. Popa University of Medicine and Pharmacy, 700115 Iasi, Romania; alina-diana.panainte@umfiasi.ro; 5 Department of Radiology, County Clinical Emergency Hospital, 720224 Suceava, Romania; mihaicreteanu@gmail.com

**Keywords:** paclitaxel, drug-eluting stent, paclitaxel carriers, controlled drug release, nano-formulations

## Abstract

Oncology and cardiology use paclitaxel, a powerful cell-division inhibitor. Paclitaxel-eluting coated metal stents in oncology prevent tumor growth by modulating proliferation, apoptosis, and angiogenesis. Paclitaxel-eluting stents reduce in-stent restenosis and maintain arterial patency in cardiology. Paclitaxel encapsulated in liposomes improves targeted administration, pharmacokinetics, and therapeutic efficacy. Liposome-loaded paclitaxel has a stronger anti-tumor effect than standard formulations. Paclitaxel plus sirolimus in a stent may synergistically prevent restenosis and inhibit tumors. Liposomal formulations with supplemental medications offer promising prospects for improving therapeutic accuracy and efficacy. Liposomal paclitaxel enhances targeted drug delivery, which may improve cancer treatment and cardiovascular treatments such as drug-eluting stents and drug-coated balloons in interventional radiology. Advanced delivery technologies have changed paclitaxel drug delivery methods for therapeutic use by overcoming its low solubility and systemic toxicity. Nanocrystals and liposomes enable high drug loading and precise targeted delivery, and, by eliminating toxic solvents such as Cremophor EL, they improve the safety and efficacy of cancer therapy.

## 1. Introduction

Paclitaxel is one of the most important natural-product-derived anticancer agents used in clinical practice. Originally isolated from the bark of Taxus brevifolia, paclitaxel became an established treatment for multiple solid malignancies, including breast, ovarian, lung, gastric, and pancreatic cancers [1,2,3]. Its antineoplastic activity results primarily from binding to β-tubulin and stabilizing microtubules, thereby suppressing normal microtubule dynamics, disrupting mitotic spindle function, and inhibiting cell division [3]. These pharmacological properties have established paclitaxel as a major component of systemic anticancer therapy.

Beyond oncology, the potent antiproliferative activity of paclitaxel has enabled its use in cardiovascular medicine. Local delivery through drug-eluting stents (DESs) and drug-coated balloons (DCBs) inhibits vascular smooth muscle cell proliferation and neointimal hyperplasia, thereby reducing restenosis following endovascular intervention [4,5]. Paclitaxel consequently represents an unusual example of a pharmacologically established compound used both for systemic anticancer treatment and for localized vascular therapy.

Despite its therapeutic efficacy, paclitaxel presents major pharmaceutical challenges because of its pronounced hydrophobicity and extremely poor aqueous solubility. Conventional intravenous paclitaxel therefore requires a solubilizing vehicle containing polyoxyethylated castor oil (Cremophor EL) and ethanol. Although this formulation enables intravenous administration, Cremophor EL has been associated with hypersensitivity reactions, altered pharmacokinetics, and the requirement for premedication [6]. These limitations have provided a major impetus for the development of alternative paclitaxel formulations.

Advances in pharmaceutical technology have consequently produced multiple solvent-free or carrier-based delivery strategies with distinct structural architectures, including albumin-bound nanoparticles, liposomal systems, polymeric micelles, biodegradable polymeric nanoparticles, and nanocrystal-based formulations [7,8]. In parallel, several of these carrier platforms can be further engineered through functional strategies such as ligand-mediated targeting, stimuli-responsive release, or combination drug delivery. These platforms are designed not only to improve paclitaxel solubility but also to modify its pharmacokinetics, biodistribution, drug-release profile, tissue exposure, and toxicity. Nab-paclitaxel represents the most established example of this transition, whereas several liposomal, polymeric, and targeted systems remain at different stages of experimental or clinical development. Importantly, these platforms should not be regarded as having equivalent levels of clinical validation.

The same principle of delivery-dependent pharmacology is evident in vascular medicine, although the therapeutic objective differs substantially from that in oncology. Whereas systemic cancer therapy seeks effective drug exposure within malignant tissue, DES and DCB technologies deliver paclitaxel locally to the arterial wall to suppress the proliferative response associated with restenosis. Device coating composition, drug dose, release kinetics, tissue transfer, and vascular retention therefore become important determinants of therapeutic performance [9]. This dual application illustrates how the clinical effects of paclitaxel depend not only on its intrinsic pharmacological activity but also on the delivery strategy and biological environment in which the drug is used.

Despite substantial progress, important translational limitations remain. Nanocarrier performance may be affected by biological heterogeneity, tissue penetration, formulation stability, manufacturing reproducibility, and regulatory requirements, while vascular drug-delivery systems require optimization of local transfer, retention, coating characteristics, and vascular compatibility [10,11,12,13,14,15,16]. Moreover, clinically established formulations and devices must be clearly distinguished from emerging platforms supported predominantly by preclinical or early-phase evidence. A critical comparison of paclitaxel delivery technologies therefore requires consideration not only of their pharmaceutical design but also of their level of clinical validation and application-specific limitations.

Accordingly, the aim of this review is to critically examine current and emerging paclitaxel delivery systems across oncology and cardiovascular medicine, with particular emphasis on solvent-based formulations, albumin-bound nanoparticles, liposomal systems, polymeric micelles and other nanocarriers, as well as DES and DCB technologies. The review compares their formulation principles, mechanisms of drug delivery, pharmacokinetic and safety implications, level of clinical evidence, translational limitations, and future development. Particular attention is given to distinguishing established clinical technologies from experimental and emerging delivery platforms, thereby providing an integrated perspective on how pharmaceutical formulation and local delivery strategies shape the therapeutic use of paclitaxel.

**Figure 1:**
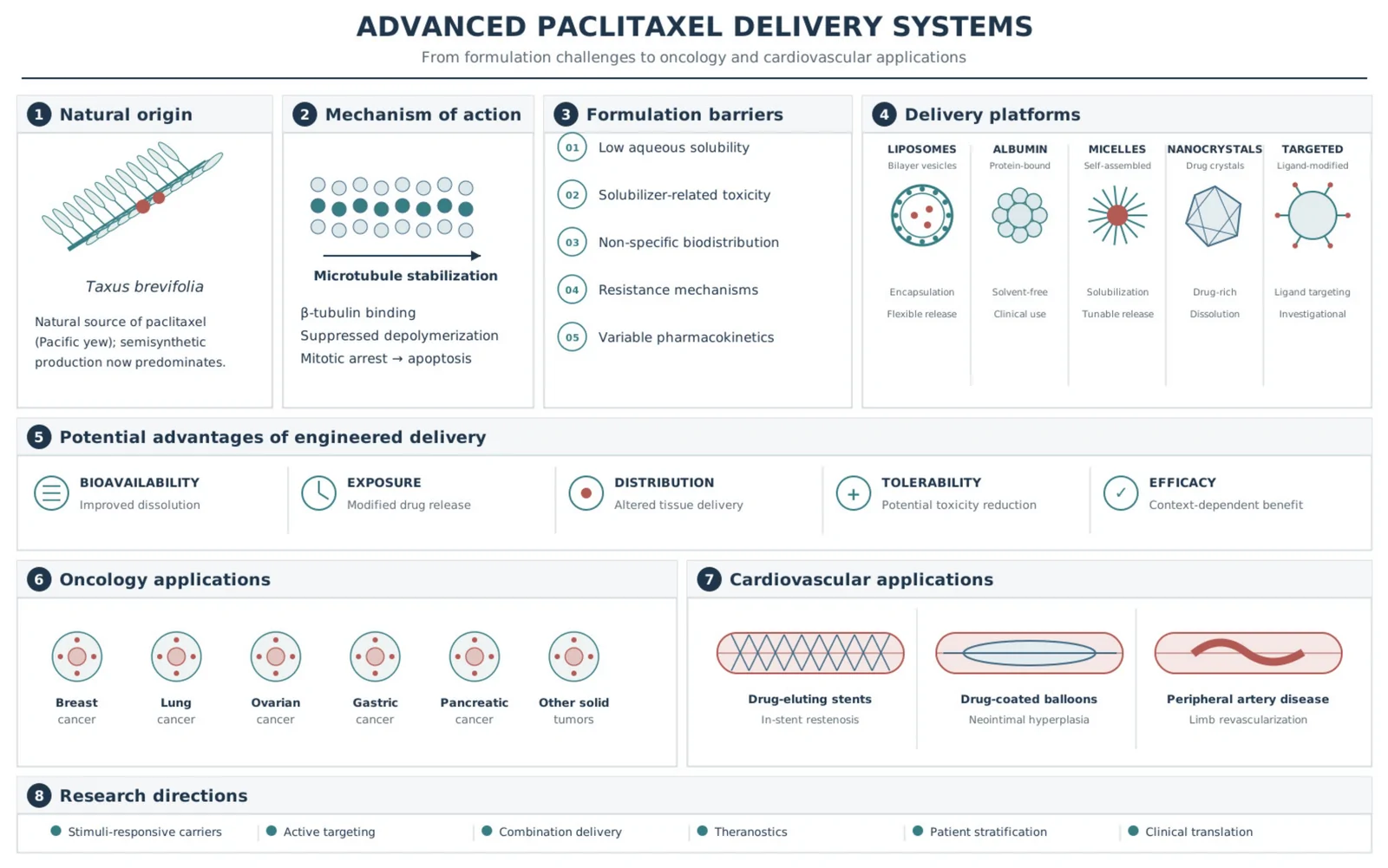
Overview of advanced paclitaxel delivery systems in oncology and cardiovascular medicine. The figure summarizes the natural origin of paclitaxel, its principal physicochemical and pharmacological limitations, and the major delivery platforms developed to improve its clinical performance. These include liposomes, albumin-bound nanoparticles, polymeric micelles, nanocrystals, and targeted nanocarriers. Their main advantages comprise enhanced solubility and bioavailability, controlled drug release, prolonged circulation, improved therapeutic efficacy, reduced systemic toxicity, and partial overcoming of multidrug resistance. The figure also highlights the principal clinical applications of paclitaxel in oncology and cardiovascular medicine, including drug-eluting stents and drug-coated balloons, together with emerging directions such as active targeting, stimuli-responsive systems, combination delivery, theranostic platforms, personalized nanomedicine, artificial intelligence-assisted formulation design, and clinical translation.

The aim of the present review is to provide a comprehensive overview of current paclitaxel delivery systems, with particular emphasis on liposomal formulations, nanoparticle-based technologies, mechanisms of bioavailability enhancement, and emerging applications in both oncology and cardiovascular medicine. Furthermore, recent advances and future perspectives in the development of innovative paclitaxel delivery platforms are critically discussed (Figure 1).

**Figure 2:**
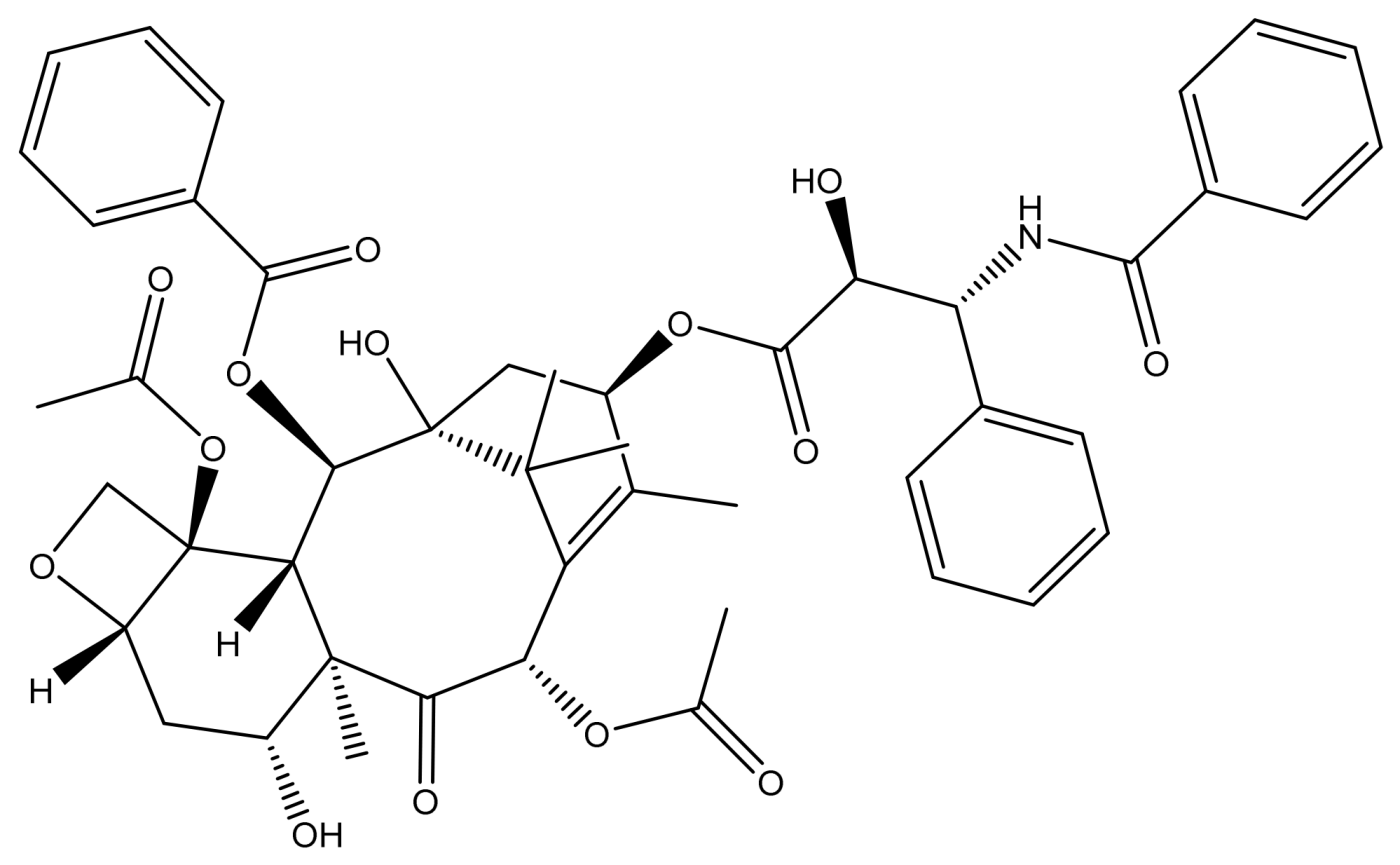
Chemical structure of paclitaxel.

Paclitaxel is one of the most important natural-product-derived anticancer agents used in clinical practice. Originally isolated from the bark of *Taxus brevifolia* during the U.S. National Cancer Institute natural-product screening program, paclitaxel was subsequently developed into an established treatment for multiple solid malignancies [1,2,3,17]. Structurally, paclitaxel is a highly lipophilic taxane with a complex diterpenoid core and an essential side chain, structural characteristics that contribute to both its pharmacological activity and its challenging pharmaceutical properties [17,18,19]. Its antineoplastic activity results primarily from binding to β-tubulin and stabilizing microtubules, thereby suppressing normal microtubule dynamics, disrupting mitotic spindle function, and inhibiting cell division (Figure 2) [3,20].

Paclitaxel exerts its principal pharmacological effect through binding to β-tubulin in assembled microtubules, promoting microtubule stabilization and suppressing normal microtubule dynamics. The resulting disruption of mitotic spindle function leads to cell-cycle arrest and contributes to its antineoplastic activity [20]. Its clinical use is nevertheless constrained by dose-dependent toxicities, particularly myelosuppression and peripheral neuropathy, while the conventional Cremophor EL-containing formulation introduces additional concerns related to hypersensitivity reactions [21,22,23]. Paclitaxel is predominantly metabolized through CYP2C8, with CYP3A4 also contributing to its metabolism, and its disposition can additionally be influenced by drug-transport mechanisms such as P-glycoprotein. These pharmacological and toxicity characteristics have provided an additional rationale for developing delivery systems that modify drug exposure while reducing formulation-related toxicity.

## 2. Paclitaxel Formulations and Drug-Delivery Systems

The clinical development of paclitaxel has been closely linked to formulation strategies capable of overcoming its extremely poor aqueous solubility while maintaining therapeutically effective drug exposure. Over time, delivery systems have evolved from solvent-based intravenous formulations toward structurally distinct platforms including albumin-bound nanoparticles, liposomal systems, polymeric micelles, biodegradable polymeric nanoparticles, nanocrystals, and other nanotechnology-based formulations. These approaches differ not only in carrier architecture and composition but also in their effects on drug solubilization, release, pharmacokinetics, biodistribution, tolerability, and clinical applicability [20].

Rather than considering formulation and bioavailability enhancement as separate topics, the principal paclitaxel delivery systems are discussed below according to carrier type, together with the mechanisms by which each platform modifies drug delivery.

The contemporary paclitaxel formulation landscape encompasses technologies with markedly different levels of maturity, ranging from established solvent-based and albumin-bound products to regionally available formulations, clinically investigated delivery systems, and predominantly preclinical targeted or multifunctional nanocarriers. These categories should therefore be compared not only according to pharmaceutical performance but also according to the strength of their clinical evidence.

### 2.1. Conventional Solvent-Based Paclitaxel

The conventional intravenous formulation of paclitaxel was developed to overcome the drug’s very low aqueous solubility. Paclitaxel is solubilized in a non-aqueous vehicle containing polyoxyethylated castor oil (Cremophor EL) and dehydrated ethanol and is subsequently diluted before intravenous infusion. Although this formulation enabled the clinical use of paclitaxel, Cremophor EL is associated with clinically relevant hypersensitivity reactions and requires corticosteroid and antihistamine premedication [24].

Cremophor EL can also influence paclitaxel disposition through the formation of micellar structures in plasma, thereby altering the fraction of freely available drugs and contributing to nonlinear pharmacokinetic behavior. These limitations provided an important rationale for the development of solvent-free formulations designed to preserve paclitaxel activity while reducing vehicle-related toxicity.

### 2.2. Albumin-Bound Nanoparticle Paclitaxel

Nanoparticle albumin-bound paclitaxel (nab-paclitaxel; Abraxane^®^) represents one of the most clinically established solvent-free alternatives to conventional paclitaxel. In this formulation, paclitaxel is associated with human serum albumin nanoparticles and administered as an intravenous suspension without Cremophor EL. Elimination of the solvent reduces solvent-associated hypersensitivity concerns and removes the requirement for routine corticosteroid premedication while allowing a shorter infusion time [25].

Albumin also functions as a biological carrier and modifies the disposition of paclitaxel. Albumin-associated transport mechanisms may contribute to drug movement across endothelial barriers and influence tissue distribution. Clinically, nab-paclitaxel has demonstrated that modification of the delivery vehicle can substantially alter the administration and tolerability profile of paclitaxel without changing its fundamental antimitotic mechanism [25].

However, the biological mechanisms proposed to explain enhanced tumor delivery should be interpreted cautiously. Carrier-mediated transport and tumor accumulation depend on tumor type, vascular characteristics, and microenvironmental factors, and therefore, should not be assumed to provide uniform targeting across all malignancies.

### 2.3. Liposomal Paclitaxel Formulations

Liposomal delivery represents a distinct strategy for incorporating hydrophobic paclitaxel into phospholipid-based vesicles. Paclitaxel can associate with the hydrophobic domains of the lipid bilayer, allowing preparation of aqueous dispersions without the conventional Cremophor EL vehicle. Liposomal composition commonly includes phosphatidylcholine and cholesterol, while PEGylated phospholipids such as DSPE-PEG may be incorporated to improve colloidal stability and modify interactions with the mononuclear phagocyte system [26,27].

Encapsulation can alter paclitaxel biodistribution and reduce immediate exposure to the free drug. PEGylation may additionally prolong systemic circulation by reducing opsonization and rapid clearance. These properties provide the rationale for investigating liposomal paclitaxel as a means of modifying tissue exposure and potentially improving tolerability relative to conventional solvent-based formulations [27].

The enhanced permeability and retention (EPR) effect has traditionally been proposed as one mechanism contributing to the preferential accumulation of nanoscale drug carriers in tumors. In preclinical models, increased vascular permeability together with impaired lymphatic drainage can favor the retention of liposomes and other nanoparticles within tumor tissue. However, the magnitude of this effect is highly variable and depends on tumor type, vascular architecture and permeability, stromal density, extracellular matrix composition, lymphatic function, particle characteristics, and other features of the tumor microenvironment. Consequently, EPR-mediated accumulation should be regarded as a context-dependent phenomenon rather than a universally reliable mechanism of tumor targeting in humans.

Importantly, evidence of increased nanoparticle accumulation in experimental tumor models should not be considered equivalent to demonstrated clinical superiority. Liposomal encapsulation can modify paclitaxel solubility, circulation behavior, biodistribution, and exposure to free drug, but these pharmaceutical and pharmacokinetic advantages do not necessarily translate into greater tumor selectivity or improved clinical outcomes in every tumor type. Clinical benefit must therefore be established independently for each formulation and indication rather than inferred from the EPR mechanism alone.

Importantly, liposomal paclitaxel should be distinguished from other nanoscale paclitaxel formulations with different carrier architectures. In particular, Genexol-PM is a polymeric micelle formulation and not a liposomal formulation [26]. This distinction is relevant when comparing delivery mechanisms and pharmaceutical properties across carrier systems.

### 2.4. Polymeric Micellar Paclitaxel

Polymeric micelles are self-assembled nanoscale structures formed from amphiphilic block copolymers. Their hydrophobic core can incorporate poorly water-soluble compounds such as paclitaxel, while the hydrophilic corona contributes to dispersion stability in aqueous environments. Commonly investigated polymers include PEG-based block copolymers such as PEG-PLA and related amphiphilic systems.

Genexol-PM is a clinically investigated Cremophor-free polymeric micelle formulation of paclitaxel and represents an important example of this approach [26,28]. Unlike liposomes, which are based on phospholipid bilayers, polymeric micelles rely on the self-assembly of amphiphilic polymers. This structural distinction influences drug incorporation, carrier stability, release characteristics, and manufacturing requirements.

Polymeric micelles can substantially increase the apparent aqueous solubility of paclitaxel and may permit modulation of drug-release behavior through changes in polymer composition. Nevertheless, their performance remains formulation-dependent, and stability after dilution, drug release, manufacturing reproducibility, and clinical validation must be considered when comparing them with more established delivery systems.

### 2.5. Biodegradable Polymeric Nanoparticles

Biodegradable polymeric carriers provide another strategy for sustained and controlled paclitaxel delivery. Poly(lactic-co-glycolic acid) (PLGA) is among the most extensively investigated polymers because its degradation behavior and drug-release characteristics can be modified through polymer molecular weight, lactide-to-glycolide ratio, particle characteristics, and formulation conditions [29].

Paclitaxel-loaded PLGA systems can protect the incorporated drug and provide release over extended periods as the polymer matrix undergoes hydrolytic degradation. This tunability makes biodegradable nanoparticles attractive for experimental controlled-delivery applications. However, the advantages observed in preclinical systems must be balanced against challenges related to drug loading, release reproducibility, manufacturing scale-up, and regulatory characterization [29].

### 2.6. Nanocrystal-Based Paclitaxel

Paclitaxel nanocrystals represent a comparatively carrier-minimized approach in which the drug itself constitutes most of the nanoparticle mass. Reducing paclitaxel to the nanoscale increases the effective surface area available for dissolution and can improve dispersion of the highly hydrophobic compound without requiring a large proportion of additional carrier material.

This approach offers the potential for high drug loading and relatively simple carrier composition. Nevertheless, nanocrystal performance depends strongly on particle size, surface stabilization, physical stability, dissolution behavior, and interactions with biological fluids. Surface modification and stabilization strategies are therefore important determinants of their pharmaceutical performance [30].

### 2.7. Engineering of Paclitaxel Delivery Systems: Biofunctionalization and Targeting

Biofunctionalization and active targeting should be considered functional engineering strategies rather than independent structural classes of paclitaxel carriers. These approaches extend conventional drug-delivery platforms by modifying the surfaces of liposomes, polymeric nanoparticles, micelles, or other carriers with molecules that interact with specific cellular or vascular targets. Investigated ligands include folate, transferrin, antibodies directed toward receptors such as HER2, and peptides containing the Arg-Gly-Asp (RGD) motif, which can interact with integrins associated with angiogenic vasculature.

Surface ligands are frequently attached through functionalized PEG chains or related chemical linkers. Their principal role is to promote molecular recognition and, where appropriate, receptor-mediated cellular internalization after the carrier reaches the target tissue. Active targeting should therefore be distinguished from passive tissue accumulation: ligand functionalization does not necessarily guarantee increased delivery of the entire administered nanoparticle dose to a tumor.

The effectiveness of biofunctionalization depends on receptor density and heterogeneity, ligand density and orientation, carrier size and surface properties, steric accessibility, protein-corona formation, and stability of the ligand–carrier linkage. These variables can substantially influence targeting efficiency and must be optimized for each delivery system.

Although targeted paclitaxel carriers offer considerable experimental potential, their level of clinical validation is heterogeneous. Many remain predominantly preclinical or at an early translational stage. Their potential advantages should therefore be distinguished from the established clinical evidence supporting conventional paclitaxel and nab-paclitaxel.

Importantly, incorporation of paclitaxel into appropriately designed delivery systems may also improve its in vitro selectivity toward malignant cells. For example, paclitaxel-loaded silver nanoparticles (AgNPs-PTX) were evaluated against MCF-7 breast cancer, A549 lung cancer, and C6 glioma cells using WI-38 fibroblasts as the non-malignant reference. Compared with the free paclitaxel, AgNPs-PTX increased the selectivity index (SI) from 2.29 to 4.17 in MCF-7 cells, from 2.53 to 6.53 in A549 cells, and from 2.08 to 4.96 in C6 cells, corresponding to approximately 1.8-, 2.6-, and 2.4-fold increases, respectively. These findings provide quantitative evidence that incorporation of paclitaxel into a drug-delivery platform can increase the differential cytotoxicity between malignant and non-malignant cells. However, such improvements are formulation- and cell-model-dependent and should not be extrapolated to other nanocarrier systems without direct comparative evaluation [31].

A similar principle has been demonstrated with targeted liposomal delivery. Folate-functionalized, pH-sensitive paclitaxel liposomes showed a significantly higher selectivity index than both non-targeted liposomal paclitaxel and free paclitaxel in experimental breast-cancer models, supporting the concept that carrier engineering and molecular targeting may further increase tumor-versus-normal-cell selectivity [32].

### 2.8. Alternative Routes and Emerging Formulations

Although intravenous administration remains central to paclitaxel therapy, alternative delivery routes have also been investigated to overcome the limitations imposed by poor aqueous solubility, intestinal metabolism, and transporter-mediated efflux. Oral delivery is particularly challenging because paclitaxel is a substrate for P-glycoprotein and undergoes presystemic metabolism, resulting in low and variable oral bioavailability.

Strategies developed to improve oral exposure include lipid-based systems, self-microemulsifying drug-delivery systems (SMEDDSs), and pharmacological inhibition of intestinal efflux transport. These approaches illustrate that formulation design can be used not only to modify intravenous paclitaxel delivery but also to address biological barriers associated with alternative administration routes [13,33].

More broadly, the expanding range of paclitaxel formulations demonstrates that the term “advanced delivery system” encompasses technologies with very different compositions, mechanisms, and levels of clinical maturity. Direct comparison therefore requires consideration of the carrier architecture, mechanism of drug incorporation and release, pharmacokinetic consequences, safety profile, manufacturing requirements, and strength of clinical evidence. The principal characteristics of the major paclitaxel formulation strategies are summarized in Table 1, emphasizing the relationship between carrier system, key excipients, functional role, and the principal pharmaceutical considerations associated with each approach.

Although paclitaxel’s list of cancer targets is growing, it is now used to treat metastatic ovarian, non-small cell lung, breast, and AIDS-related Kaposi’s sarcoma.

Paclitaxel also inhibits proliferation. Neointimal hyperplasia, angiographic restenosis, and repeated revascularization in percutaneous coronary procedures are significantly reduced by paclitaxel stent elution [34].

Its mechanism of action relies on selective binding to the β-subunit of tubulin in assembled microtubules. Paclitaxel stabilizes polymerized microtubules and prevents depolymerization, whereas Vinca alkaloids inhibit tubulin polymerization. The disruption of mitotic spindle dynamics caused by this stabilization results in dose-dependent anti-angiogenic and immunomodulatory effects in addition to mitotic inhibition. Thus, it contributes to the prevention of tumor growth by inhibiting endothelial cell migration and vascular endothelial growth factor (VEGF)-mediated angiogenesis at low doses.

Crucially, efflux transporters such as P-glycoprotein (P-gp) have a significant impact on the intracellular concentration of paclitaxel. P-gp overexpression is linked to multidrug resistance (MDR) in a number of tumor types. By encouraging endocytic uptake pathways rather than passive diffusion, sophisticated drug delivery devices partially get around this resistance mechanism [35].

Paclitaxel is being researched for additional diseases that need microtubule stabilization and avoidance of angiogenesis and cell proliferation, such as psoriasis, one of the most puzzling and persistent skin ailments. Paclitaxel is being studied as a treatment for Alzheimer’s and Parkinson’s disease, which could increase its therapeutic uses.

Although the same active compound is used in both fields, paclitaxel delivery in oncology and cardiovascular medicine addresses fundamentally different pharmacological and technological requirements. In oncology, delivery strategies primarily aim to optimize systemic pharmacokinetics, tumor biodistribution, tissue penetration, intracellular drug exposure, and treatment-related toxicity. Accordingly, carrier architecture, circulation time, tumor accumulation, and biological heterogeneity are major determinants of therapeutic performance. In cardiovascular medicine, by contrast, paclitaxel is predominantly administered locally through drug-eluting stents (DESs) and drug-coated balloons (DCBs). In this setting, therapeutic performance depends primarily on coating characteristics, drug dose, release and transfer kinetics, arterial-wall uptake and retention, and vascular compatibility. Because these two applications involve fundamentally different delivery objectives, the oncological and cardiovascular uses of paclitaxel are discussed separately in the following sections.

### 2.9. Side Effects of Paclitaxel Administration

#### 2.9.1. Hypersensitivity Reactions

Paclitaxel’s early development was impeded by significant hypersensitivity reactions that reached 30% in some experiments. Initial findings blamed histamine and other vasoactive chemicals for hypersensitive reactions.

Most people with type 1 hypersensitivity reactions suffer from dyspnea, urticaria, and hypotension. Chest or back pain, excessive sweating, and pruritus were also prevalent. Paclitaxel-induced hypersensitivity reactions are considerably reduced by oral dexamethasone premedication at 12 and 6 h before infusion. Although this method has not been shown universally successful, patients with major hypersensitivity reactions rechallenged with paclitaxel after high corticosteroid doses have had no recurrences.

#### 2.9.2. Neuropathies

Paclitaxel can cause peripheral neuropathy with sensory indications of paresthesia and numbness in a glove and stocking distribution. Loss of sensation including proprioception, vibration, pinprick and temperature is also often symmetrical.

Symptoms are generally seen between 24 and 72 h after paclitaxel treatment with higher dosages of the drug (>250 mg/m^2^), but frequently after numerous courses at normal dosage protocols (<200 mg/m^2^). Severe neurotoxicity is rare even at standard doses and in patients previously exposed to neurotoxic drugs such as cisplatin. If neurotoxicity is severe, the medicine cannot be given in high doses for up to 24 h.

Autonomic and motor dysfunction can also be a concern of paclitaxel therapy, especially in patients with preexisting neuropathies due to alcoholism, diabetes mellitus and other pathological disorders. Optic nerve may also be involved (e.g., scintillating scotoma). History of past chemotherapy and timing of administration are critical factors in development of nab-paclitaxel-induced sensory neuropathy.

Transient myalgia is common, and usually occurs 2 to 5 days after therapy at dosages > 170 mg/m^2^. Insidious myopathy has been reported with high dosages of paclitaxel, often in combination with cisplatin stated above [21].

#### 2.9.3. Other Common Side-Effects

One of the main harmful consequences of paclitaxel is non-cumulative neutropenia, or low neutrophil count, which means that the immature hematopoietic cells are not permanently destroyed. The medicine may also lower the quantity of erythrocytes, leading to anemia, and thrombocytes, leading to bruising and bleeding. Normal heart rhythm may be altered, most often transiently and asymptomatically as bradycardia. Cardiac monitoring is recommended in individuals with ventricular dysfunction and in those who may not tolerate the potential bradyarrhythmia of the medicine. Gastrointestinal symptoms are also common, especially at larger doses, and include diarrhea and vomiting. These conditions require at least 2 L of hydration every day. Leukemia patients are at particular risk for mucositis and disintegration of the mucosal barrier.

Inflammation at the injection site, along the vein and sites of drug extravasation from the blood vessel and inflammatory skin reactions following this may infrequently occur. Paclitaxel is substantially metabolized by liver enzymes (CYP2C8 and CYP3A4). The co-administration of inhibitors or inducers of certain enzymes can drastically alter drug levels and require careful control of the prescription. The main problem of side effects is that new formulations, solvent free formulations, are associated with a greatly reduced risk of severe hypersensitivity reactions and the burden of premedication compared to previous formulations (Table 2 and Table 3) [22].

## 3. Mechanisms of Increasing Paclitaxel Bioavailability

Paclitaxel is very hydrophobic and poorly soluble in water and therefore affects the systemic availability. The drug is encapsulated in the lipid bilayer of liposomes which overcomes the problem of solubility and allows the preparation of a stable concentrated iv. solution without the use of harmful cosolvents. Original solvent: Cremophor EL is initially necessary for iv. solubility but causes severe hypersensitivity reactions and dramatically reduces oral bioavailability by micelle formation, which traps the drug and affects P-gp efflux pumps. Innovation in industry evolved from solvent-based systems to albumin-bound nanoparticles (e.g., Abraxane) and liposomal formulations (e.g., Lipusu and Genexol-PM) to improve the bioavailability and safety. These options can remove toxic solvents, improve solubility, lengthen circulation duration of the medicine and allow passive targeting of tumors using the EPR effect. Nanomedicine totally remodels paclitaxel and eliminates toxicity. The medicine in a nanocarrier is shielded against fast metabolism and deactivation in plasma or in the liver. The liposomes also aid to overcome P-glycoprotein efflux pumps in cell membranes, which generally pump free paclitaxel out of cells and severely limit its absorption and effectiveness. Beyond improvements in solubility, pharmacokinetics, and drug delivery, the biocompatibility of paclitaxel formulations should be evaluated using complementary preclinical in vivo evidence. Studies in mice and rats can provide relevant information on systemic tolerability, body-weight changes, hematological toxicity, organ-specific toxicity, and histopathological alterations following administration of paclitaxel-loaded delivery systems. Such evidence is particularly important because reduced carrier- or solvent-related toxicity cannot be inferred solely from physicochemical properties or in vitro cytotoxicity. Accordingly, the principal pharmaceutical advantages of advanced paclitaxel formulations should be considered together with their available preclinical safety and biocompatibility data (Table 4) [36].

Beyond improvements in solubility, pharmacokinetics, and drug delivery, the safety and biocompatibility of paclitaxel formulations should be supported by preclinical in vivo evidence. Comparative studies in mice and rats indicate that selected liposomal and polymeric micellar formulations can modify the toxicity and tolerability profile of paclitaxel relative to conventional solvent-based formulations, although these effects remain formulation-dependent [37,38,39]. Representative findings are summarized in Table 4.

Importantly, improved pharmaceutical performance does not necessarily imply improved biocompatibility. Preclinical evaluation of paclitaxel delivery systems should therefore assess the complete formulation, including both the active drug and the carrier, using relevant systemic and organ-specific toxicity endpoints. Comparative in vivo studies are particularly informative when they include free or conventionally formulated paclitaxel as a reference, because they allow potential reductions in formulation-related toxicity to be distinguished from the intrinsic toxicological effects of paclitaxel.

Standard paclitaxel clears quickly from the bloodstream. Liposomal formulations, and especially those coated by the polyethylene glycol (PEGylated) escape the mononuclear phagocytic system in the liver and spleen, which results in a great increase in the biological half-life (from a few hours to over 50 h in certain studies) Tumors generally have blood arteries that are “leaky”, with larger gaps than those in healthy tissue, and often have poor lymphatic drainage. Liposomes of a typical size (60–150 nm) can passively penetrate these gaps and concentrate in the tumor microenvironment [40].

### Antibody–Drug Conjugates (ADCs)

This is considered one of the most modern targeted oncology therapies. Paclitaxel (or taxane derivatives) is chemically linked to a specific monoclonal antibody. The antibody exclusively targets the receptors on tumor cells (acting like a guided missile), and paclitaxel is released only inside the tumor, completely shielding the body’s healthy cells from the systemic effects of chemotherapy.

Achieving effective oral paclitaxel treatment remains difficult, requiring specialized systems (SMEDDS) often co-administered with P-gp inhibitors like encequidar to counteract intestinal efflux mechanisms [33].

## 4. Liposomal Formulations for Paclitaxel-Comparative Approach and Future Perspectives

In summary, liposomes act as protective, highly effective transport systems that fundamentally change how paclitaxel behaves in the body, ensuring more of the drug reaches the tumor site efficiently and safely. Suprageneric versions of paclitaxel were developed, such as nanoparticle albumin-bound paclitaxel (Abraxis Oncology’s Abraxane^®^) and polyglutamate paclitaxel (Cell Therapeutics’ Xyotax^®^). Their advantages are in terms of drug delivery and a lower number of side effects (Table 5) [27].

### 4.1. Advanced Nanocarrier Engineering Strategies

While the first-generation liposomal paclitaxel formulations focused on solubility and toxicity, second- and third-generation nanocarriers seek to improve tumor selectivity, intracellular trafficking, and controlled release kinetics. 

Active targeting strategies require surface conjugation of ligands that bind to tumor-specific receptors. Commonly targeted moieties include:

- Folate targets—Folate receptor-α in ovarian and breast cancer.
- Transferrin targets—Overexpressed transferrin receptors in proliferative cells.
- Liposomes functionalized with HER2 antibodies (trastuzumab).
- RGD peptides that bind to integrins in angiogenic vasculature.

Conjugation commonly attaches ligands to PEG termini by maleimide-thiol chemistry or carbodiimide crosslinking.

Active targeting improves receptor-mediated endocytosis, intracellular drug accumulation and decreases off-target toxicity. However, clinical translation has been hampered by immunogenicity issues, receptor variability, and complex manufacturing needs.

Albumin-Based Nanoparticle Systems are the right approach for paclitaxel regarding liposomal formulation (Table 4). Nanoparticle albumin-bound paclitaxel (nab-paclitaxel) is a clinically proven solvent-free formulation.

#### 4.1.1. Mechanism for Enhanced Tumor Uptake

Albumin interacts with endothelial cells’ gp60 receptors, resulting in caveolae-mediated transcytosis. Additionally, tumor-associated protein SPARC (secreted protein acidic and rich in cysteine) has high affinity for albumin, boosting accumulation inside the tumor interstitium.

Compared with solvent-based paclitaxel, the pharmacokinetic profile of…Abraxane presents a higher maximum tolerated dose (300 mg/m^2^ vs. ~240 mg/m^2^), linear pharmacokinetics, reduced infusion time and no need for steroid premedication. Clinical trials show better response rates in metastatic breast cancer [41].

#### 4.1.2. Biofunctionalization of Paclitaxel Drug-Delivery Systems

Biofunctionalization represents an important strategy for extending paclitaxel nanocarriers beyond passive drug encapsulation toward biologically interactive delivery systems. In this context, the surface of liposomes, polymeric nanoparticles, micelles, or other nanocarriers can be modified with molecular ligands designed to interact with receptors or molecular structures expressed by tumor cells, tumor-associated endothelium, or other target tissues. The principal objective is to enhance cellular recognition and internalization while limiting nonspecific drug exposure.

Several targeting strategies have been investigated for paclitaxel delivery systems. Folate-functionalized carriers exploit the increased expression of folate receptor-α in selected malignancies, whereas transferrin-based systems target transferrin receptors associated with proliferating cells. Antibody-functionalized carriers, including HER2-directed approaches, provide another strategy for receptor-mediated targeting. Peptides containing the Arg-Gly-Asp (RGD) motif have also been investigated because of their affinity for integrins associated with angiogenic vasculature. These ligands can be incorporated onto carrier surfaces, frequently through functionalized PEG chains, to facilitate receptor recognition while maintaining colloidal stability.

Biofunctionalization may influence several stages of drug delivery. Following systemic administration, the functionalized carrier must remain sufficiently stable in the circulation, reach the target tissue, interact with the intended molecular receptor, and subsequently promote cellular association or receptor-mediated internalization. Therefore, active targeting should not be interpreted simply as increased accumulation of nanoparticles within the target tissue. In many systems, its principal advantage may instead involve improved cellular recognition and internalization after the carrier has reached the pathological microenvironment.

The effectiveness of biofunctionalization nevertheless depends on multiple biological and pharmaceutical variables, including receptor density and heterogeneity, ligand density and orientation, carrier size and surface properties, steric accessibility of the targeting ligand, protein-corona formation, and stability of the ligand–carrier linkage. Excessive ligand density may also alter circulation behavior or increase nonspecific interactions. Consequently, optimization requires balancing targeting capacity with the physicochemical and pharmacokinetic properties of the nanocarrier.

Importantly, the biological rationale for active targeting does not imply equivalent clinical validation across platforms. Many biofunctionalized paclitaxel delivery systems remain predominantly experimental, and their translation may be limited by tumor heterogeneity, immunogenicity, manufacturing complexity, reproducibility, and regulatory requirements. Accordingly, biofunctionalization should presently be considered a promising strategy for improving the biological interaction of paclitaxel delivery systems rather than a universally established mechanism for superior clinical targeting.

An important distinction among paclitaxel delivery systems concerns their level of clinical development. The platforms discussed in this review should not be interpreted as having equivalent translational status. Some formulations, particularly conventional solvent-based paclitaxel and nab-paclitaxel, are established clinical products, whereas other liposomal, polymeric, actively targeted, stimuli-responsive, and multifunctional systems range from regionally available formulations to clinical-stage candidates and predominantly preclinical technologies. Accordingly, developmental status is specified for individual formulations where reliable product-specific evidence is available rather than being generalized to an entire carrier class.

### 4.2. Polymeric Micelle and PLGA-Based Systems

Polymeric micelles are self-assembled amphiphilic block copolymers containing a hydrophobic core with paclitaxel encapsulation, along with stabilization through the hydrophilic corona. Common polymers that are used are PEG-b-PLA and PEG-b-PCL.

#### Poly(Lactic-co-Glycolic Acid) (PLGA)

PLGA systems enable biodegradable, controlled release via hydrolytic polymer degradation. The release kinetics can be adjusted by controlling the lactide/glycolide ratio, the molecular weight and polymer crystallinity. These devices produce medication release profiles that last several days or weeks. This technique improves tumor transport over passive EPR buildup [29].

Recent developments in paclitaxel delivery illustrate a progressive diversification from conventional solvent-based formulations toward clinically established nanocarriers and increasingly sophisticated experimental platforms. Importantly, these technologies have reached markedly different stages of translation. Nab-paclitaxel represents an established solvent-free albumin-bound formulation with extensive clinical use, while Lipusu provides an example of a liposomal paclitaxel formulation used clinically in specific regions. Genexol-PM represents a distinct polymeric micelle platform that has progressed through clinical development and reached clinical use in several countries. In parallel, biodegradable polymeric nanoparticles, paclitaxel nanocrystals, ligand-functionalized carriers, and stimuli-responsive or multifunctional systems are being investigated to improve drug loading, controlled release, tumor recognition, intracellular delivery, or combination therapy; however, many of these approaches remain predominantly preclinical or at an early translational stage. Thus, recent progress in paclitaxel delivery should be interpreted not only in terms of technological sophistication but also according to the maturity of the supporting clinical evidence. Table 6 summarizes representative examples and compares their clinical or regulatory status, principal applications, evidence level, delivery advantages, toxicity considerations, manufacturing complexity, and major translational limitations.

## 5. Key Insights into Paclitaxel DES and Drug-Coated Devices

Recent development of cardiovascular paclitaxel delivery has followed a different trajectory from systemic oncology formulations. Current innovation is centered on optimization of device coatings, polymer-free delivery, DCB technologies, drug-transfer efficiency, and tissue retention, together with emerging hybrid or dual-drug approaches. These developments are discussed below using representative DES and DCB examples, while distinguishing established vascular technologies from experimental platforms.

### 5.1. Paclitaxel Drug-Eluting Stents (DESs)

Innovations are exploring new coatings with programmable pharmacokinetic release capabilities to enhance the medication delivery patterns. The cytostatic properties, rather than the cytotoxic properties, of paclitaxel make it an attractive agent for vascular therapy.

To summarize, new findings suggest that while paclitaxel DES remains a viable option for peripheral applications, advancements in coronary intervention have largely favored limus-based therapies due to their improved efficacy in the specific coronary microenvironment, and that the technology is generally moving toward polymer-free solutions or balloon-based delivery systems.

Regarding restenosis, paclitaxel inhibits smooth muscle cell migration and proliferation at nanomolar doses.

Polymer-based DES is the first-generation of paclitaxel DES, including:

- Stainless steel scaffolding.
- Durable polymer coating (e.g., SIBS or PBMA).
- Embedded paclitaxel.

**Figure 3:**
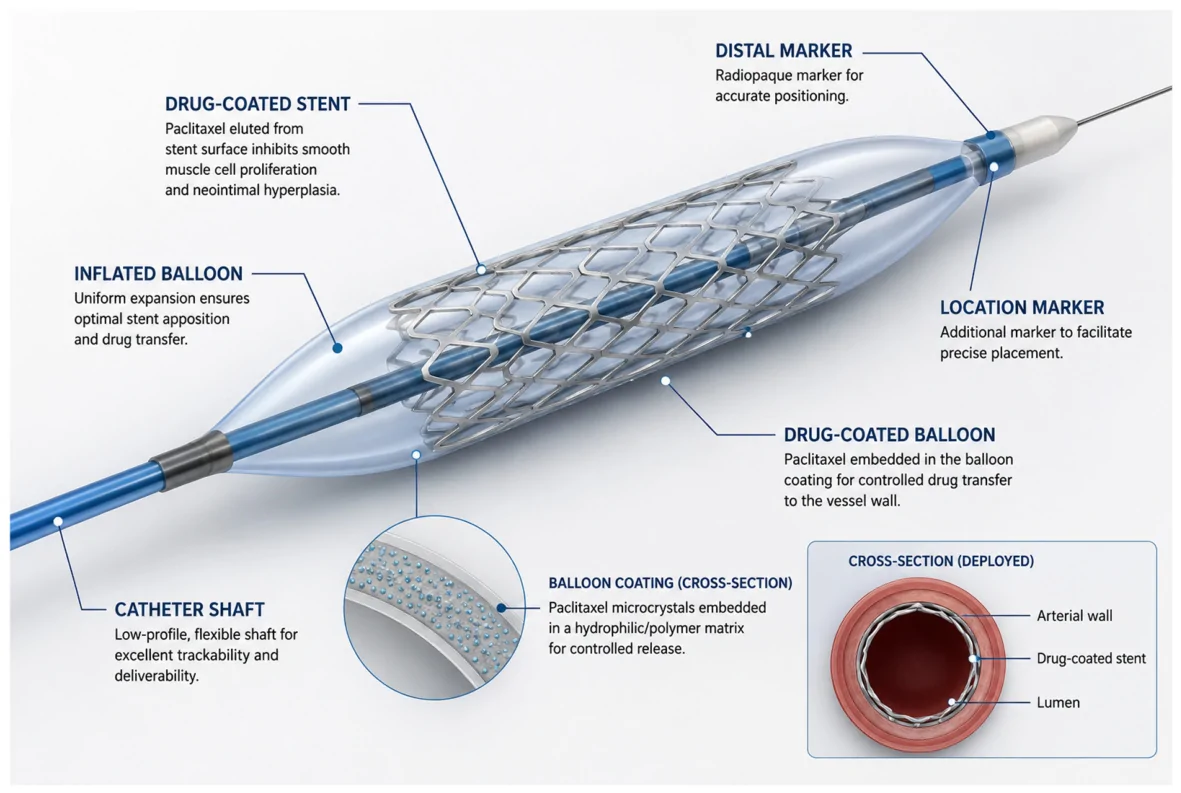
Drug Eluting Stent (DES) with Paclitaxel. Created in Leonardo AI (https://www.leonardo.ai/ai-upscaler).

Drug release follows diffusion-controlled kinetics, so is important to take in consideration polymer biocompatibility, chronic inflammatory responses, and delayed endothelial healing (Figure 3) [42].

A key insight is paclitaxel’s efficacy is location-dependent. It is still an effective antiproliferative drug for the treatment of PAD, and evidence confirms its safety and long-term efficacy in preventing restenosis.

### 5.2. Shift to Polymer-Free Designs

Recent advances in paclitaxel drug-eluting stents (DESs) seem to be heading toward polymer-free designs and a major surge in the usage of drug-coated balloons (DCBs) as an alternative. Following percutaneous coronary intervention (PCI), endothelial injury stimulates smooth muscle cell proliferation, extracellular matrix deposition, neointimal hyperplasia and restenosis. Paclitaxel inhibits smooth muscle cell migration and proliferation at nanomolar doses.

While paclitaxel remains effective for peripheral artery disease (PAD), new data emphasizes its limited efficacy in the specific hypoxic environment of coronary lesions compared to “limus” drugs (sirolimus and derivatives).

A major trend in paclitaxel stent technology is the development of polymer-free coatings. This approach aims to deliver the drug to the arterial wall without leaving behind a permanent polymer (a “leave nothing behind” construction). Polymer-free DESs lower the risk of late stent thrombosis, polymer-free methods use by microporous stent surfaces, microcrystalline paclitaxel coatings and direct drug deposition. These technologies are designed to administer drugs without the use of long-term polymers. Paclitaxel DCBs are quite effective in peripheral arterial disease (PAD), especially in the femoropopliteal arteries. Safety and efficacy of paclitaxel for PAD were confirmed in a 2024 5-year data release, with no significant difference in all-cause mortality compared with non-coated devices in several large studies, addressing previous safety concerns raised in meta-analyses.

Clinical experiments are testing microdrop spray crystallization of polymer-free paclitaxel-coated stents [43].

### 5.3. Rise in Drug-Coated Balloons (DCBs)

A major alternative that has been developed is the use of DCBs that distribute paclitaxel without leaving a permanent metallic scaffold. DCBs have been particularly important in the treatment of in-stent restenosis (ISR) and small-artery disease. Long-term results from trials such as ISAR-DESIRE 3 showed no difference in mortality endpoints between paclitaxel-eluting balloons and paclitaxel-eluting stents for ISR, providing evidence that DCBs are a safe and effective “leave nothing behind” treatment strategy.

Drug-Coated Balloons (DCBs) mechanism for drug transfer takes place during the balloon inflation, with the following steps: (a) paclitaxel is administered in high concentration to the vessel wall; (b) fast tissue absorption occurs due to high drug lipophilicity; (c) sustained action is provided by keeping the drug in the intima.

The main coating excipients include urea, iopromide and shellac, which improve medication transfer efficiency [44].

Paclitaxel has been substantially displaced in coronary arteries by limus-family medicines due to greater performance in hypoxic coronary plaque microenvironments. Sirolimus or its derivatives (everolimus, zotarolimus) are commonly used in second- and third-generation DES for treatment of coronary artery disease (CAD). New research reveals that sirolimus is more effective at blocking proliferation in the low-oxygen (hypoxic) environment of advanced atherosclerotic plaques, a situation that greatly reduces the efficacy of paclitaxel. The difference in mechanism makes limus medicines better suited to prevent restenosis in the coronary arteries [45].

Sirolimus and its analogs have a low transfer rate, as compared with paclitaxel, and are kept in the tissues for a lengthy period. At present, a number of local balloon-based delivery approaches of sirolimus and its variants have been suggested. In the pre-clinical model, the biological effect of the zotamox coating compared with the paclitaxel coating was validated [46].

Currently, paclitaxel is still the main choice for DCB, and the typical dose is 2~3.5 μg/mm^2^. Variable coating formulations and coating methods may result in variable pharmacokinetic properties, which is one of the main considerations for optimal drug delivery. The interplay of drug dosage, preparation, release kinetics and lesion appeared to be the key to the vascular response after DCB treatment.

The simple lesion suggests a hemispheric saccule or a non-compliant balloon of the same diameter of the artery. In case of suspicion of vessel filling or possible insufficient size and difficult balloon delivery, the starting point is a smaller balloon which reassesses the vessel size following the use of vasodilators. If the conventional semicompliant balloon fails to dilate, a high-pressure noncompliant balloon or cutting balloon should be considered [47].

### 5.4. Emerging Dual-Drug and Hybrid Drug-Coated Technologies

The established clinical role of paclitaxel in vascular intervention should be distinguished from emerging dual-drug delivery concepts. Paclitaxel-based DCBs are clinically established in peripheral vascular intervention and have also been evaluated in selected coronary applications, whereas contemporary coronary DES predominantly use sirolimus or related limus-family agents. These technologies are supported by substantially greater clinical experience than currently available hybrid paclitaxel/sirolimus platforms [42,43,44,45,46,47].

Dual-drug delivery has been proposed as a strategy for combining agents with different physicochemical and pharmacological characteristics within a single vascular delivery platform. In principle, paclitaxel provides rapid tissue uptake because of its high lipophilicity, whereas sirolimus and related agents act through inhibition of mTOR-dependent cell proliferation. Combining these agents could therefore offer complementary delivery or biological characteristics. However, a plausible mechanistic rationale should not be interpreted as evidence of pharmacological synergy.

At present, paclitaxel/sirolimus dual-drug DCBs and related hybrid systems should be regarded as emerging or developmental technologies rather than established clinical alternatives. Available evidence is considerably less mature than that supporting contemporary single-drug paclitaxel DCBs or limus-based coronary technologies. Preclinical or early developmental observations may support further investigation, but they do not establish improved vascular healing, reduced restenosis, or superior clinical outcomes.

Accordingly, claims of a fixed optimal paclitaxel-to-sirolimus ratio, substantial dose reduction through synergistic action, or superior endothelial healing should not be generalized without platform-specific experimental evidence and appropriate comparative clinical validation. The clinical value of dual-drug delivery will ultimately require adequately powered studies comparing these technologies with contemporary single-drug DCBs and established DES strategies using clinically relevant endpoints.

Thus, hybrid drug delivery represents an interesting direction in vascular-device development, but its potential advantages should currently be considered hypothesis-generating rather than clinically established.

The level of clinical evidence supporting paclitaxel delivery systems varies substantially and cannot be inferred from carrier type alone. Some advanced formulations have reached routine or region-specific clinical use, whereas others remain at different stages of clinical development or predominantly preclinical investigation. For example, Genexol-PM has undergone randomized Phase III evaluation against conventional paclitaxel, while Lipusu has been evaluated in clinical studies and is used as a liposomal paclitaxel formulation in China. In contrast, many actively targeted, stimuli-responsive, and multifunctional nanocarriers remain primarily experimental. Accordingly, Table 7 compares the principal platforms not only according to pharmaceutical characteristics but also according to the maturity of their supporting evidence.

Paclitaxel acts as a “penetration vector.” Due to its extreme lipophilicity, it leads the way into the arterial wall and rapidly halts the proliferation of smooth muscle cells (which cause vessel blockage). Supported by sirolimus, paclitaxel is effective at a dose 13 times lower than standard formulations, eliminating the risk of vessel wall necrosis (Table 6) [48].

Older excipients (the carrier substances that helped paclitaxel detach from the balloon) could generate micro-emboli. New generations of DCBs use paclitaxel nanocrystals or phospholipid micro-reservoirs. These optimize drug transfer deep into the tunica media of the artery without losing the active substance into the bloodstream.

## 6. Discussion

### 6.1. Comparative Appraisal of Paclitaxel Delivery Platforms

The development of paclitaxel delivery systems has been driven largely by a practical pharmaceutical problem: an effective but highly hydrophobic drug required a formulation capable of achieving clinically useful exposure without introducing unacceptable toxicity. Early solvent-based formulations addressed the solubility problem but introduced limitations related to Cremophor EL, including hypersensitivity reactions and altered pharmacokinetic behavior [23,49]. Subsequent formulations have therefore focused not only on solubilizing paclitaxel but also on controlling how the drug is distributed, released, and retained in target tissues.

This evolution has produced several delivery strategies with different strengths rather than a single universally superior platform. Albumin-bound paclitaxel is among the most clinically established solvent-free alternatives and avoids Cremophor EL while allowing shorter infusion schedules and reducing the need for premedication [50,51]. Liposomal formulations offer a different set of advantages, including modification of drug distribution and considerable flexibility in particle composition and surface engineering. Their performance, however, remains sensitive to lipid composition, particle size, encapsulation efficiency, stability, and the biological characteristics of the target tissue [52]. Polymeric micelles and biodegradable nanoparticles provide even greater flexibility in controlling drug loading and release, but this versatility is accompanied by manufacturing, reproducibility, and regulatory challenges [53].

These differences are important when comparing delivery technologies. Improvements in solubility, circulation time, or drug loading do not necessarily translate into superior clinical efficacy. A formulation that performs well in a controlled experimental system must still demonstrate adequate safety, reproducibility, manufacturability, and clinical benefit. The relevant comparison is therefore not simply whether one carrier produces a favorable pharmaceutical parameter, but whether its overall profile offers a meaningful advantage for a particular clinical indication [54,55].

The field is increasingly moving toward multifunctional systems capable of combining controlled release with targeting, intracellular delivery, or diagnostic functionality [56,57]. However, the level of evidence supporting these technologies is highly heterogeneous. Established formulations should therefore be distinguished from experimental nanocarriers whose advantages remain predominantly preclinical. This distinction is particularly important when interpreting emerging targeting and multifunctional strategies.

### 6.2. Translational Implications in Oncology

In oncology, the central challenge of paclitaxel delivery is achieving therapeutically effective tumor exposure while limiting systemic and formulation-related toxicity. Consequently, the clinically relevant performance of a delivery platform depends on systemic pharmacokinetics, biodistribution, tumor penetration, intracellular drug availability, and the biological heterogeneity of the tumor microenvironment. Although the pharmacological target of paclitaxel remains unchanged, modification of its carrier can substantially influence systemic exposure, tissue distribution, infusion requirements, and treatment-related toxicity [58]. The clinical relevance of a delivery system therefore depends on the interaction among the drug, carrier architecture, tumor biology, and patient characteristics.

One of the clearest benefits of solvent-free formulations has been the reduction in toxicities associated with the vehicle rather than with paclitaxel itself. Removing Cremophor EL can reduce hypersensitivity-related concerns and simplify premedication and administration [59]. This represents a clinically meaningful advantage, but it should be distinguished from claims that a carrier inherently increases tumor selectivity or antitumor efficacy.

This distinction is particularly relevant to nanoparticle-mediated tumor accumulation. Nanocarriers may modify paclitaxel biodistribution and can increase drug exposure within tumors under favorable experimental conditions [60]. Nevertheless, tumor vascular permeability, stromal architecture, extracellular matrix density, and molecular transport pathways vary considerably between tumors and between patients [61]. Consequently, passive accumulation mechanisms, including the enhanced permeability and retention effect, should not be regarded as uniformly effective in human malignancies.

Active targeting and biofunctionalization may provide an additional level of selectivity by promoting interactions with defined cellular or vascular targets. Surface-functionalized nanoparticles, ligand-mediated systems, and other targeted carriers are therefore attractive approaches for improving cellular recognition and internalization [62]. Yet their clinical value depends on factors such as target expression, intratumoral heterogeneity, carrier accessibility, and manufacturing reproducibility. For many of these systems, compelling preclinical activity has not yet been matched by an equivalent level of clinical validation.

The most realistic near-term role of advanced paclitaxel delivery in oncology may therefore be the selective use of formulations whose pharmacokinetic or safety advantages address a defined clinical need rather than the assumption that increasingly complex carriers will necessarily produce better outcomes. Future development will require closer integration of formulation science with tumor biology and patient selection [30].

A particular limitation concerns the translation of the EPR concept from experimental models to human cancer. Much of the mechanistic evidence supporting passive nanoparticle accumulation originates from preclinical tumor models, whereas human tumors show substantially greater heterogeneity in vascular permeability, perfusion, stromal organization, extracellular matrix density, and lymphatic drainage. These differences can produce marked variability in nanoparticle delivery both between tumor types and among patients with the same malignancy. EPR-mediated accumulation should therefore be interpreted as one potential contributor to nanocarrier biodistribution rather than as a predictable form of passive tumor targeting. From a clinical perspective, improved pharmacokinetics or altered biodistribution should be distinguished from demonstrated improvements in tumor response, survival, or treatment-related toxicity.

### 6.3. Cardiovascular Applications and Local Vascular Drug Delivery

In cardiovascular medicine, the delivery problem is fundamentally different from that encountered in oncology. Paclitaxel is not intended to achieve sustained systemic exposure but rather to produce a controlled local antiproliferative effect within the arterial wall. Drug-eluting stents (DESs) and drug-coated balloons (DCBs) therefore aim to provide sufficient local paclitaxel exposure to inhibit vascular smooth muscle cell proliferation and neointimal hyperplasia while minimizing systemic drug loss [63].

Consequently, the performance of cardiovascular paclitaxel delivery systems depends not only on the pharmacological activity of the drug but also on drug dose, coating composition and morphology, release or transfer kinetics, drug loss during device delivery, arterial-wall uptake and retention, lesion preparation, and vascular compatibility [64]. These parameters are particularly important for DCBs, where drug transfer occurs during a short period of balloon–arterial wall contact, whereas DESs provide drug release from a coating associated with a permanent vascular scaffold. This explains why apparently similar paclitaxel-coated devices cannot necessarily be considered pharmacologically interchangeable. This explains why apparently similar paclitaxel-coated devices cannot necessarily be considered pharmacologically interchangeable.

The role of paclitaxel also differs between vascular territories. In peripheral arterial interventions, paclitaxel-based technologies continue to have an established role, supported by contemporary evidence regarding efficacy and long-term safety [65]. In coronary DES, by contrast, clinical practice has largely shifted toward sirolimus and related limus-family agents. This difference illustrates a broader principle: the optimal delivery platform is determined by the biological and procedural environment in which the drug is used, rather than by the pharmacological properties of the active compound alone.

The move toward polymer-free coatings and DCB-based strategies reflects an effort to achieve effective local drug delivery while minimizing permanent material within the vessel [66]. Emerging hybrid or dual-drug approaches extend this concept further, but they should be interpreted cautiously. At present, mechanistic rationale and preclinical performance should not be presented as equivalent to demonstrated clinical synergy or superiority. Robust comparative clinical evidence will be required before such platforms can be considered established alternatives.

Thus, the cardiovascular experience with paclitaxel illustrates the close relationship between pharmacology and device engineering. For local vascular therapy, therapeutic performance results from the interaction among the drug, its carrier or coating, the device, lesion characteristics, and the vascular bed itself [67,68].

### 6.4. Current Limitations and Translational Challenges

Despite the large number of experimental paclitaxel delivery systems described in the literature, relatively few have progressed to routine clinical use. This gap between preclinical innovation and clinical adoption remains one of the central challenges in nanomedicine [69]. Improved drug loading, targeting, or release characteristics are valuable experimental findings, but they are insufficient by themselves to establish clinical superiority.

Biological heterogeneity is a major contributor to this translational gap. Tumors differ in vascular permeability, stromal composition, receptor expression, immune environment, and tissue architecture, all of which can influence nanocarrier distribution and drug penetration [70]. A formulation optimized in one experimental model may therefore perform differently in another tumor type or patient population. This variability is especially relevant to delivery strategies that depend heavily on passive accumulation or a single molecular target.

Manufacturing presents a second major barrier. Complex nanocarriers require reproducible control of particle size, drug loading, surface properties, release kinetics, and stability. Increasing carrier complexity may improve functionality while simultaneously making scale-up, quality control, regulatory characterization, and batch-to-batch reproducibility more difficult [71]. For this reason, technological sophistication should not itself be considered evidence of translational superiority.

Safety must also be evaluated at the level of the complete formulation. Removing Cremophor EL can eliminate important vehicle-associated toxicities, but novel polymers, surface modifications, targeting ligands, and other carrier components introduce their own biological considerations. Long-term safety data are limited for many emerging systems, particularly those that remain at the preclinical stage [72].

Similar considerations apply to cardiovascular devices. Coating durability, drug loss during delivery, arterial-wall transfer, release kinetics, and vascular compatibility can all influence clinical performance [73]. Economic feasibility is also relevant across both oncology and cardiovascular medicine; a technologically sophisticated platform must ultimately provide sufficient clinical benefit to justify manufacturing complexity and cost [74].

Taken together, these limitations suggest that the main challenge is no longer simply to create more elaborate paclitaxel carriers. The more difficult task is to identify which technological modifications provide reproducible and clinically meaningful advantages and to validate them in appropriately designed comparative studies [69,71,75].

### 6.5. Future Perspectives

Future paclitaxel delivery systems are likely to become more biologically responsive rather than merely more complex. Active targeting, biomimetic carriers, stimuli-responsive release, and combination delivery are being investigated as ways of adapting drug exposure more closely to the characteristics of the target tissue [76,77,78,79]. These strategies are scientifically attractive, but their development should be guided by demonstrable biological need and clinically relevant endpoints.

Biofunctionalized and biomimetic systems are of particular interest because they may improve interactions between carriers and target cells. Antibodies, peptides, aptamers, small-molecule ligands, cell-membrane coatings, and extracellular-vesicle-inspired systems have all been explored as approaches to improve cellular uptake or biological compatibility [77]. Their eventual clinical value, however, will depend on whether these additional design features produce reproducible benefits that justify greater manufacturing and regulatory complexity.

Stimuli-responsive platforms provide another potential route toward more controlled delivery. Systems triggered by pH, enzymes, reactive oxygen species, temperature, ultrasound, magnetic fields, or light may permit drug release to be restricted more closely in space and time [78]. Similarly, co-delivery of paclitaxel with nucleic acids, targeted agents, or other therapeutic compounds could address multiple mechanisms of treatment resistance simultaneously [79]. Most of these strategies nevertheless remain developmental and require stronger translational evidence before conclusions regarding clinical superiority can be drawn.

Computational methods may also become increasingly useful in formulation development. Machine-learning and mechanistic modeling approaches could assist in screening formulation variables, predicting release behavior, and identifying relationships between carrier properties and pharmacokinetic performance [80]. Their most immediate value may lie in reducing the experimental search space and improving formulation design rather than replacing experimental or clinical validation.

Clinical evidence does not currently support the assumption that liposomal or nanoparticle paclitaxel provides universally superior tumor targeting compared with conventional or albumin-bound paclitaxel. Randomized and comparative clinical studies of liposomal paclitaxel have generally demonstrated antitumor activity comparable to conventional paclitaxel, with potential advantages in formulation-related tolerability rather than consistently superior efficacy. Moreover, the EPR effect is markedly heterogeneous in human tumors and is influenced by vascular permeability, perfusion, stromal composition, extracellular matrix density, lymphatic function, and tumor type. Consequently, improved nanoparticle accumulation observed in preclinical models should not be equated with predictable passive targeting or superior clinical outcomes in patients.

Ultimately, successful translation will depend on a balance between innovation and practicality. Manufacturing at scale, regulatory standardization, long-term safety, cost-effectiveness, and randomized clinical evidence remain necessary regardless of how sophisticated a delivery platform becomes [81]. The future of paclitaxel delivery should therefore be judged not by technological complexity alone, but by whether a formulation provides a reproducible improvement in drug exposure, safety, efficacy, or patient outcome.

In this context, the continued evolution of paclitaxel provides a useful model for drug-delivery research more broadly. The same molecule has moved from solvent-based systemic administration to albumin-based nanoparticles, experimental nanocarriers, and highly localized vascular devices. This history demonstrates that the therapeutic performance of a drug can be substantially shaped by how and where it is delivered [82,83,84,85,86,87].

## 7. Materials and Methods

This narrative review was conducted through a comprehensive literature search focusing on the pharmaceutical development, clinical applications, and emerging delivery technologies of paclitaxel. Relevant publications were identified through electronic databases including PubMed, Scopus, Web of Science, and Google Scholar.

The literature search was performed between January and February 2026 and included articles published primarily between 2000 and 2025, with particular emphasis on studies published during the last five years. Earlier landmark publications were also included when considered essential for understanding the historical development, pharmacological mechanisms, and clinical evolution of paclitaxel-based therapies.

The following keywords and combinations were used: “paclitaxel”, “liposomal paclitaxel”, “paclitaxel liposomes”, “nanoparticle albumin-bound paclitaxel”, “nab-paclitaxel”, “polymeric micelles”, “drug delivery systems”, “nanomedicine”, “drug-eluting stents”, “drug-coated balloons”, “vascular drug delivery”, “pharmacokinetics”, “bioavailability”, and “targeted therapy”.

Original research articles, clinical trials, meta-analyses, systematic reviews, and relevant regulatory or clinical reports were considered eligible for inclusion. Studies were selected based on their scientific relevance, methodological quality, and contribution to understanding the formulation strategies, pharmacological properties, therapeutic efficacy, and safety profile of paclitaxel delivery systems.

The collected literature was subsequently organized into thematic sections covering the historical development of paclitaxel, physicochemical and pharmacological characteristics, formulation technologies, liposomal and nanoparticle-based delivery systems, mechanisms of bioavailability enhancement, cardiovascular applications including drug-eluting devices, and future perspectives for advanced paclitaxel delivery platforms.

## 8. Conclusions

In 2025, paclitaxel is still an important drug because it can stabilize microtubules and stop cells from dividing, which makes it useful in both systemic cancer and localized cardiovascular intervention. However, there have been worries in the past about a “late mortality signal” in peripheral devices; most experts agree that the benefits in terms of patency and quality of life often outweigh the risks, especially with better tracking at the patient level and more modern device designs. Paclitaxel research shows that it may have antiplatelet and antithrombotic effects inside stents, which could lower the risk of local blood clots in addition to its main role of stopping cell growth.

## Figures and Tables

**Table 1:** Types of paclitaxel formulations.

Formulation Type	Primary Solubilizing/ Carrier System	Key Excipients	Excipients Functional Role	Observations
Conventional (Solvent-Based) Paclitaxel	Non-aqueous surfactant–ethanol vehicle	Polyoxyethylated castor oil (Cremophor EL); Dehydrated ethanol	- Solubilization of highly lipophilic paclitaxel; - Formation of micellar solution upon dilution	- Associated with hypersensitivity reactions; - Altered pharmacokinetics due to micellar entrapment
Albumin-Bound Nanoparticle Paclitaxel	Protein-stabilized nanoparticles	Human serum albumin (HSA); typically no synthetic surfactants.	- Drug binding and nanoparticle stabilization; - Facilitated endothelial transcytosis via albumin-mediated pathways	- Removes Cremophor EL; - Improved tolerability profile
Polymeric Micellar Paclitaxel	Amphiphilic block copolymer micelles	Poly(ethylene glycol)-block-poly(lactic acid) (PEG-PLA); Poly(ethylene glycol)-block-poly(aspartate), or similar amphiphilic polymers	- Self-assembly into nanoscale micelles; - Hydrophobic core solubilizes paclitaxel; - Hydrophilic corona enhances stability	- Enhanced solubility; - Prolonged circulation depending on polymer composition; - Controlled release properties; - Scalable, but formulation-sensitive
Liposomal Paclitaxel	Phospholipid bilayer vesicles	Phosphatidylcholine; Cholesterol; PEGylated phospholipids (e.g., DSPE-PEG)	- Encapsulation within lipid bilayer or membrane; - Membrane stabilization; - Steric stabilization (PEGylation)	- Modified biodistribution; - Potential reduction in systemic toxicity.

**Table 2:** Side Effects For Paclitaxel. WBC = white blood cells; RBC = red blood cells.

System/Category	Common Side Effects	Management/Notes
Blood & Immune System	Neutropenia (low WBC count) and Infection	Requires frequent monitoring and preventative measures (hygiene and avoiding crowds) are essential.
	Anemia (low RBC count)	Can cause fatigue and weakness and may require blood transfusions in severe cases.
	Thrombocytopenia	Increased risk of bruising and bleeding; avoid NSAIDs and rigorous activity.
	Hypersensitivity Reactions	Flushing, rash, chest pain, and difficulty breathing. Premedication is necessary for solvent-based forms (Taxol).
Nervous System	Peripheral Neuropathy (numbness, tingling, pain in hands/feet)	A dose-limiting toxicity; usually reversible after treatment, but can persist.
	Myalgia/Arthralgia	Can be managed with pain relievers like acetaminophen.
Gastrointestinal	Nausea and Vomiting	Anti-nausea medications are typically prescribed; dietary changes can help.
Skin & Hair	Alopecia	Hair typically regrows after treatment completion.
	Injection Site Reactions	Redness, swelling, pain, or hardening at the iv. site. Report signs of extravasation immediately.
Cardiovascular	Bradycardia and Hypotension	Monitored during infusion; severe events are rare but possible.
Liver	Liver Toxicity (elevated liver enzymes)	Monitored via regular blood tests.

**Table 3:** Drug interactions and potential side effects [24,33].

Drugs	Potential Effect	Recommendation
CYP2C8 & CYP3A4 Inhibitors (Ketoconazole, Erythromycin, Clarithromycin, Fluoxetine, Grapefruit juice)	Increased paclitaxel levels; higher risk of toxicity.	Avoid or use alternate drugs/monitor closely.
CYP2C8 & CYP3A4 Inducers (Rifampin, Phenytoin, Carbamazepine, St. John’s Wort)	Decreased paclitaxel levels; potentially reduced efficacy.	Avoid or monitor closely.
P-glycoprotein (P-gp) inhibitors (Cyclosporin, Atorvastatin, Valspodar)	Increased intracellular accumulation of paclitaxel; enhanced neurotoxicity.	Use with caution and monitor for neuropathy.
Live Vaccines	Immunosuppression from paclitaxel may reduce vaccine effectiveness or cause infection.	Avoid live vaccines during treatment.
Doxorubicin	Increased risk of toxicity if given concurrently; reduced doxorubicin clearance.	A minimum 24-h interval between administrations is recommended.
Palifermin	Increased severity and duration of oral mucositis.	Do not administer within 24 h of paclitaxel.

**Table 4:** Pharmaceutical advantages and representative preclinical biocompatibility evidence for advanced paclitaxel delivery systems.

Paclitaxel Delivery System	Main Pharmaceutical Advantages	Preclinical Model	Comparator	Representative Preclinical Safety/Biocompatibility Findings	Ref.
Liposome-encapsulated paclitaxel	Cremophor EL-free delivery; improved aqueous dispersion; modified pharmacokinetics and tissue distribution	Normal mice	Conventional paclitaxel	Repeated administration at 32.5 mg/kg resulted in no mortality with liposome-encapsulated paclitaxel, whereas conventional paclitaxel produced 100% mortality under the same dosing regimen. The liposomal formulation also altered paclitaxel pharmacokinetics and tissue distribution.	[37]
Polymeric micellar paclitaxel (Pm-Pac)	Solvent-free delivery; improved apparent solubility; potential modulation of systemic exposure and drug disposition	Healthy Sprague–Dawley rats	Solvent-based paclitaxel	Toxicity occurred at approximately 2–3-fold higher doses with Pm-Pac than with solvent-based paclitaxel. Safety evaluation included body weight, survival, peripheral neuropathy, hematological parameters, and histopathology. Reduced brain and liver injury was observed, although potential male reproductive toxicity remained a formulation-specific safety consideration.	[38]
Elastic liposomal paclitaxel	Cremophor EL-free formulation; improved drug incorporation; increased tolerated dose and reduced formulation-related toxicity	Mice	Marketed Cremophor EL-based paclitaxel	The formulation showed an approximately six-fold higher maximum tolerated dose. The reported LD50 was 141.6 mg/kg versus 16.7 mg/kg for the conventional formulation, together with reduced hemolytic toxicity.	[39]

Abbreviations: Pm-Pac, polymeric micellar paclitaxel; LD50, median lethal dose. Preclinical safety findings are formulation- and model-specific and should not be interpreted as evidence of equivalent clinical safety or superior therapeutic efficacy.

**Table 5:** Industrial formulations for iv. administration of paclitaxel.

Feature	Taxol^®^ (and Generics)	Abraxane^®^ (Nab-Paclitaxel)	Liposomal Formulations (e.g., Lipusu^®^, Genexol-PM^®^)
Formulation Type	Solvent-based i.v. solution	Albumin-bound nanoparticles (i.v. suspension)	Lipid vesicle encapsulation (i.v. suspension)
Excipients	Cremophor EL, dehydrated ethanol, citric acid	Human serum albumin	Phospholipids (e.g., phosphatidylcholine)
Premedication Required?	Yes (corticosteroids, antihistamines due to Cremophor EL)	No (Cremophor-free)	No (Cremophor-free, reduced toxicity)
Maximum Tolerated Dose	Lower (~240 mg/m^2^)	Higher (~300 mg/m^2^)	Higher (reduced systemic toxicity allows higher dose)
Hypersensitivity Risk	Significant risk	Very low risk	Very low risk
Availability	Globally approved	Globally approved	Approved in specific regions (e.g., China, South Korea)

**Table 6:** Comparative clinical and translational status of major paclitaxel delivery platforms.

Delivery Platform	Clinical/Regulatory Status	Principal Application	Evidence Level	Major Delivery/PK Advantage	Main Toxicity Consideration	Manufacturing Complexity	Principal Translational Limitation
Conventional solvent-based paclitaxel	Established clinical use	Multiple solid tumors	Extensive clinical use	Established dosing and clinical experience	Cremophor EL-associated hypersensitivity; paclitaxel-related neuropathy and myelosuppression	Low–moderate	Solvent-related toxicity and premedication requirements
Nab-paclitaxel	Approved; established clinical use	Breast, pancreatic, NSCLC and other defined indications	Phase III/extensive clinical use	Cremophor-free delivery; altered disposition; shorter infusion	Paclitaxel-related neuropathy and myelosuppression remain relevant	Moderate	Cost and indication-specific benefit; not universally superior to conventional paclitaxel
Liposomal paclitaxel—Lipusu	Regionally approved/clinically used, particularly China	Breast, ovarian and NSCLC	Clinical use and randomized clinical evidence	Cremophor-free lipid delivery; modified biodistribution	Paclitaxel-related toxicity remains possible despite removal of Cremophor	Moderate–high	Regional availability; generalizability of clinical evidence and variable tumor accumulation
Polymeric micelle paclitaxel—Genexol-PM	Approved in South Korea and several other countries	Breast cancer, NSCLC, ovarian cancer	Phase I–III and clinical use	Cremophor-free micellar delivery; allows higher paclitaxel dosing in studied regimens	Neuropathy, myalgia and neutropenia remain dose-limiting	Moderate–high	Regulatory status differs geographically; clinical benefit cannot be generalized to all micellar systems
Other polymeric/biodegradable nanoparticles	Mostly investigational	Primarily oncology	Predominantly preclinical; selected clinical-stage systems	Tunable release and carrier properties	Carrier- and formulation-dependent	High	Scale-up, reproducibility, regulatory characterization
Paclitaxel nanocrystals	Mainly investigational	Oncology; multiple experimental routes	Predominantly preclinical/early translational	High drug loading; reduced carrier burden; enhanced dissolution	Depends on particle characteristics and exposure	Moderate–high	Physical stability, reproducible particle control, limited clinical validation
Actively targeted/biofunctionalized carriers	Experimental	Precision oncology	Predominantly preclinical	Receptor-mediated recognition/internalization	Potential off-target interactions and carrier-related effects	High	Receptor heterogeneity, protein corona, manufacturing complexity, limited clinical evidence
Stimuli-responsive/multifunctional systems	Experimental	Precision/combination oncology	Preclinical	Conditional release and multiple functionalities	Platform-specific and incompletely characterized	Very high	Complexity, reproducibility, safety, regulatory burden, lack of comparative clinical evidence

**Table 7:** Comparative characteristics, clinical applications, advantages, and limitations of paclitaxel-eluting stents, drug-coated balloons, and emerging dual-drug vascular delivery systems.

Feature/Criterion	Paclitaxel Drug-Eluting Stents (DESs)	Paclitaxel Drug-Coated Balloons (DCBs)	Dual-Drug/Hybrid Technology (e.g., Paclitaxel + Sirolimus)
Mechanical Scaffold	Permanent metallic frame keeps the vessel open.	No permanent scaffold left behind (“leave nothing behind”).	No permanent scaffold left behind (applied on a balloon platform).
Primary Mechanism	Provides rigid structural support and continuous, slow drug release.	Delivers a rapid, high-dose drug transfer directly into the vessel wall.	Synergistic effect: Combines fast-acting lipophilic drug transfer with long-term anti-inflammatory drug action.
Active Drug(s)	Paclitaxel (1st Gen)/Everolimus or Zotarolimus (Modern Gen).	Paclitaxel only (highly lipophilic).	Combination: Usually Paclitaxel + Sirolimus simultaneously.
Vessel Motion	Inhibits natural vasomotion; acts as a rigid cage.	Preserves natural vessel anatomy and physiological vasomotion.	Preserves natural vasomotion while drastically reducing tissue irritation.
Key Advantages	Prevents acute elastic recoil. Gold standard for large, complex de novo lesions.	Zero risk of long-term stent thrombosis. Shorter DAPT duration required.	Faster endothelial healing than standalone paclitaxel. Lower restenosis rates in complex anatomy.
Major Limitations	Permanent foreign body. Risk of late/very late stent thrombosis.	Risk of acute elastic recoil post-inflation. Risk of coronary dissection.	Higher cost of the device. Requires very strict, flawless lesion preparation.
DAPT Duration	Long-term (6 to 12 months).	Short-term (4 weeks for stable patients).	Short-term (4 weeks), combining DCB safety with DES-like efficacy.
Primary Indications	Large de novo coronary lesions and flow-limiting dissections.	In-stent restenosis (ISR) and small-vessel disease (<2.5 mm).	Complex bifurcations, long diffuse lesions, and high-risk diabetic patients.
Key Clinical Data	TAXUS trials; ISAR-DESIRE.	BASKET-SMALL 2; ISAR-DESIRE 3.	Emerging trials on Dual-Drug DCBs (e.g., SirPlux Duo or similar hybrid matrix systems).

## Data Availability

No new data were created or analyzed in this study. Data sharing is not applicable to this article.

## Abbreviations

The following abbreviations are used in this manuscript:

ADC	Antibody–Drug Conjugate
CAD	Coronary Artery Disease
DCB	Drug-Coated Balloon
DES	Drug-Eluting Stent
DAPT	Dual Antiplatelet Therapy
DSPE-PEG	1,2-Distearoyl-sn-glycero-3-phosphoethanolamine–Polyethylene Glycol
EPR	Enhanced Permeability and Retention
FDA	Food and Drug Administration
HER2	Human Epidermal Growth Factor Receptor 2
HSA	Human Serum Albumin
ISR	In-Stent Restenosis
IV	Intravenous
MDR	Multidrug Resistance
Nab-paclitaxel	Nanoparticle Albumin-Bound Paclitaxel
PAD	Peripheral Artery Disease
PBMA	Poly(butyl methacrylate)
PCI	Percutaneous Coronary Intervention
PCL	Poly(ε-caprolactone)
PEG	Polyethylene Glycol
PEG-PLA	Poly(ethylene glycol)-block-poly(lactic acid)
P-gp	P-Glycoprotein
PLA	Poly(lactic acid)
PLGA	Poly(lactic-co-glycolic acid)
SMEDDS	Self-Microemulsifying Drug Delivery System
SIBS	Poly(styrene-block-isobutylene-block-styrene)
SPARC	Secreted Protein Acidic and Rich in Cysteine
VEGF	Vascular Endothelial Growth Factor
DDS	Drug Delivery System
PTX	Paclitaxel
